# Correction: Modifying the magnetic response of magnetotactic bacteria: incorporation of Gd and Tb ions into the magnetosome structure

**DOI:** 10.1039/d2na90036j

**Published:** 2022-05-17

**Authors:** E. M. Jefremovas, L. Gandarias, L. Marcano, A. Gacía-Prieto, I. Orue, A. Muela, M. L. Fdez-Gubieda, L. Fernández Barquín, J. Alonso

**Affiliations:** Dpto. CITIMAC, Universidad de Cantabria 39005 Santander Spain martinjel@unican.es javier.alonsomasa@unican.es; Dpto. Inmunología, Microbiología y Parasitología, Universidad del País Vasco (UPV/EHU) 48940 Leioa Spain; Helmholtz–Zentrum Berlin für Materialien und Energie Albert-Einstein-Str. 15 12489 Berlin Germany; Dpto. Electricidad y Electrónica, Universidad del País Vasco (UPV/EHU) 48940 Leioa Spain; Dpto. Física Aplicada, Universidad del País Vasco (UPV/EHU) 48013 Bilbao Spain; SGIker Medidas Magnéticas, Universidad del País Vasco (UPV/EHU) 48940 Leioa Spain; BCMaterials, Basque Center for Materials, Applications and Nanostructures, UPV/EHU Spain

## Abstract

Correction for ‘Modifying the magnetic response of magnetotactic bacteria: incorporation of Gd and Tb ions into the magnetosome structure’ by E. M. Jefremovas *et al.*, *Nanoscale Adv.*, 2022, https://doi.org/10.1039/d2na00094f.

The authors regret that an incorrect version of eqn (2) was included in the original article. The correct version of eqn (2) is presented below.2
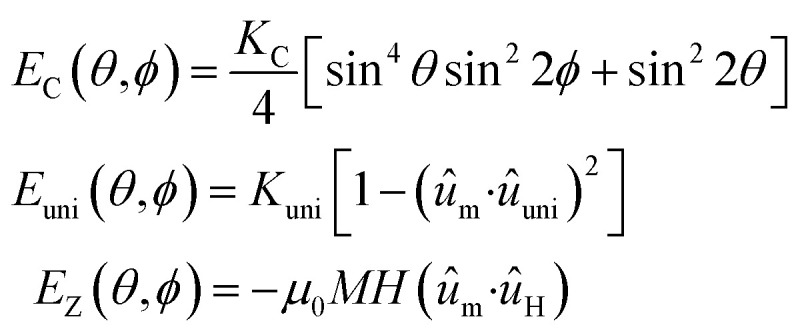


The Royal Society of Chemistry apologises for these errors and any consequent inconvenience to authors and readers.

## Supplementary Material

